# Ultrasensitive dynamic light scattering immunosensing platform for NT-proBNP detection using boronate affinity amplification

**DOI:** 10.1186/s12951-021-01224-5

**Published:** 2022-01-06

**Authors:** Jiaqi Hu, Lu Ding, Jing Chen, Jinhua Fu, Kang Zhu, Qian Guo, Xiaolin Huang, Yonghua Xiong

**Affiliations:** 1 State Key Laboratory of Food Science and Technology, School of Food Science and Technology, Nanchang University, Nanchang, 330047 People’s Republic of China; 2 Hypertension Research Institute of Jiangxi Province, Department of Cardiology, The First Affiliated Hospital of Nanchang University, Nanchang, 330006 Jiangxi People’s Republic of China; 3 Jiangxi Agricultural Technology Extension Center, Nanchang, 330046 People’s Republic of China; 4 Jiangxi-OAI Joint Research Institute, Nanchang University, Nanchang, 330047 People’s Republic of China

**Keywords:** Dynamic light scattering, Boronate affinity, Aggregation, Immunosensor, NT-proBNP

## Abstract

Herein, we reported a new dynamic light scattering (DLS) immunosensing technology for the rapid and sensitive detection of glycoprotein N-terminal pro-brain natriuretic peptide (NT-proBNP). In this design, the boronate affinity recognition based on the interaction of boronic acid ligands and *cis*-diols was introduced to amplify the nanoparticle aggregation to enable highly sensitive DLS transduction, thereby lowering the limit of detection (LOD) of the methodology. After covalently coupling with antibodies, magnetic nanoparticles (MNPs) were employed as the nanoprobes to selectively capture trace amount of NT-proBNP from complex samples and facilitate DLS signal transduction. Meanwhile, silica nanoparticles modified with phenylboronic acid (SiO_2_@PBA) were designed as the crosslinking agent to bridge the aggregation of MNPs in the presence of target NT-proBNP. Owing to the multivalent and fast affinity recognition between NT-proBNP containing *cis*-diols and SiO_2_@PBA, the developed DLS immunosensor exhibited charming advantages over traditional immunoassays, including ultrahigh sensitivity with an LOD of 7.4 fg mL^−1^, fast response time (< 20 min), and small sample consumption (1 μL). The DLS immunosensor was further characterized with good selectivity, accuracy, precision, reproducibility, and practicability. Collectively, this work demonstrated the promising application of the designed boronate affinity amplified-DLS immunosensor for field or point-of-care testing of *cis*-diol-containing molecules.

**Supplementary Information:**

The online version contains supplementary material available at 10.1186/s12951-021-01224-5.

## Highlight

1. We developed a new DLS immunosensing technology for the rapid and sensitive detection of glycoprotein NT-proBNP.
2. The boronate affinity recognition amplified nanoparticle aggregation was designed to enable highly sensitive DLS transduction.
3. The fabricated DLS immunosensor exhibited ultrahigh sensitivity with an LOD of 7.4 fg mL^−1^, fast response time (< 20 min), and small sample consumption (1 μL).
4. This boronate affinity amplified-DLS immunosensor has broad prospects for field or point-of-care testing of *cis*-diol-containing molecules.

**Supplementary Information:**

The online version contains supplementary material available at 10.1186/s12951-021-01224-5.

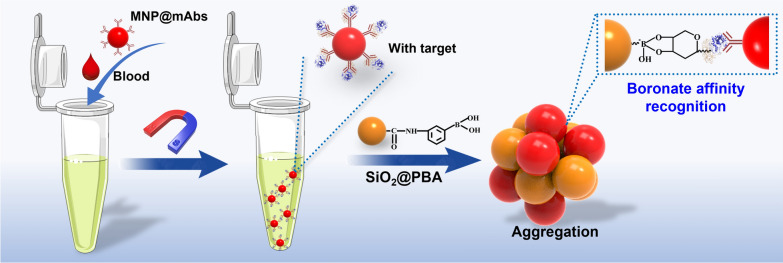

## Introduction

Heart failure (HF) is one of the most common cardiovascular diseases, which often adversely affects cardiovascular health and has become a major cause of death in humans [1]. Early diagnosis of HF contributes to timely intervention, treatment, and prognosis. N-terminal pro-brain natriuretic peptide (NT-proBNP) has been considered as a clinically recognized biomarker for early diagnosis of HF [2]. In general, the population with the NT-proBNP concentrations exceeding 300 pg mL^−1^ is high risk for HF [3]. Therefore, ultrasensitive detection for NT-proBNP is crucial for accurate clinical diagnosis and prognosis to reduce the hospitalization and mortality rates. At present, versatile immunoassay approaches have been reported to improve the determination of NT-proBNP, including colorimetric, fluorescent, electrochemical, field effect transistor, photoelectrochemical, electrochemiluminescence, and surface-enhanced Raman scattering [4–10]. Although current methods succeed in achieving the specific detection of NT-proBNP, they are still compromised by insufficient sensitivity, long response time, large sample consumption and limited use in the field of point-of-care (POC) testing.

Detecting trace target analytes in highly sensitive way usually depends on the sensitive transduction techniques and the effective signal reporting strategies. In recent years, increasing interest has been focused on exploring technologies to enhance the signal transduction for designing high-performance immunosensors [11, 12]. By coupling the strong light scattering properties of gold nanoparticles with the dynamic light scattering (DLS) technique routinely used for nanoparticle size characterization, Huo’s group has pioneered the development of a DLS-based immunosensing platform for monitoring proteins [13]. Inspired by this work, many research groups have extended DLS enhanced immunosensors for the detection of small molecules, metal ions, and microorganisms [14–16]. The currently available strategies to manipulate the light scattering signals of intensity or hydrodynamic diameter (D_H_) of nanoparticles and construct DLS immunosensors mainly involve the size change of individual nanoparticles upon target binding and the target-induced nanoparticle aggregation or self-assembly upon the antigen–antibody reaction [17–19]. However, owing to relatively limited signal fluctuations caused by target analytes based on antigen–antibody recognition, most of the reported DLS immunosensors have sensitivity levels of ng mL^−1^ [20], largely confining their direct use in response to trace amounts of NT-proBNP (pg mL^−1^).

Development of strategies to amplify the nanoparticle aggregation for improving DLS signal transduction contributes to highly sensitive detection. Due to the specific interaction of boronic acid ligands with *cis*-diols, boronate affinity materials have attracted increasing attention in many important fields, such as disease diagnosis, cell targeting, and bacterial identification and killing [21–24]. Different from the monovalent or divalent antigen–antibody interaction, the boronate affinity reaction can readily achieve the multivalent binding between boronic acid ligands and *cis*-diol-containing molecules (e.g., glycoproteins), which make them act as a promising bridge to amplify the nanoparticle aggregation [25]. Leveraging this design concept, for the first time we developed a boronate affinity amplified DLS immunosensing platform for rapid and ultrasensitive detection of trace NT-proBNP, an important serum glycoprotein marker [26]. To this end, antibody-functionalized magnetic nanoparticles (MNPs) were designed as the sensitive and specific probes to selectively capture NT-proBNP from complex samples by magnetic properties and facilitate highly sensitive DLS signal transduction using light scattering properties. In addition, phenylboronic acid modified silica nanoparticles (SiO_2_@PBA) with low scattering background signal were used as the crosslinking agent to amplify the MNP aggregation in the presence of target NT-proBNP, which can further improve the detection performances of the methodology. By virtues of the multivalent and fast boronate affinity recognition between glycoprotein NT-proBNP and SiO_2_@PBA, the developed DLS immunosensor showed the advantages of ultrahigh sensitivity (7.4 fg mL^−1^), rapid response time (< 20 min), and small sample consumption (1 μL). Besides, the selectivity, accuracy, precision, reproducibility, and practicability of this immunosensor were well demonstrated by an assay of NT-proBNP in human serum. Briefly, this work demonstrated the boronate affinity amplified DLS immunosensing strategy could detect NT-proBNP in rapid and highly sensitive manner, implying the feasibility for incorporating the nanoparticle crosslinking amplification strategy into DLS immunosensors to ultrasensitively monitor trace target analytes, even in field or at the POC.

## Materials and methods

### Regents and apparatus

Both carboxyl-functionalized MNPs (150 nm, 25 mg mL^−1^) and silica nanoparticles (SiO_2_, 100 nm, 25 mg mL^−1^) were obtained from Tianjin Baseline ChromTech Research Center (Tianjin, China). Anti-NT-proBNP monoclonal antibody (mAb) was purchased from Medix Biochemica (Espoo, Finland). Glucose, galactose, 3-aminophenylboronic acid (PBA) hydrochloride, and 1-(3-Dimethylaminopropyl)-3-ethyl-carbodiimide (EDC) were provided from Sigma-Aldrich (St. Louis, MO, USA). Serum samples used in this study were from healthy volunteers and patients who had signed informed consent forms. All experiments using human serum samples were approved by the Medical Ethics Committee of the First Affiliated Hospital of Nanchang University. All chemicals of analytical grade were provided by Sinopharm Chemical Corp (Shanghai, China) and were used without further purification.

DLS measurements for size distribution and zeta potential were conducted on a Malvern Zetasizer Nano ZSZEN3700 DLS nanoparticle analyzer (London, UK). High-resolution transmission electron microscopy (TEM) images were obtained on a JEOL JEM 2100 microscope (Tokyo, Japan). Field-emission scanning electron microscopy (SEM) images were performed using a JEOL JSM-6701F microscope (Tokyo, Japan). Fourier transform infrared (FTIR) spectrum was measured on a Thermo Fisher Nicolet iS50 infrared spectrometer (Waltham, US). Millipore water was prepared on an Elix-3 and Milli-QA water-purification system.

### Preparation of antibody modified MNP conjugates (MNP@mAb)

The conjugation of MNPs and anti-NT-proBNP mAb was conducted by using the EDC-mediated covalent coupling after electrostatic absorption. In a typical procedure, 3 μL of carboxyl functionalized MNPs (20 mg mL^−1^) were added into 200 μL of 0.01 M phosphate buffer (PB, pH 7.4), followed by the addition of 3 μg of anti-NT-proBNP mAb. After gentle stirring for 30 min at room temperature, 1.5 µL of EDC solution (1 mg mL^−1^) was added into the mixed solution. After another 30 min, the same amount of EDC was added. Finally, the resultant MNP@mAb conjugates were purified by an external magnetic field, re-dispersed in 100 µL of PB (0.01 M, pH 7.4), and stored at 4 °C until further use.

### Preparation of PBA-functionalized SiO_2_ (SiO_2_@PBA)

**Scheme 1:**
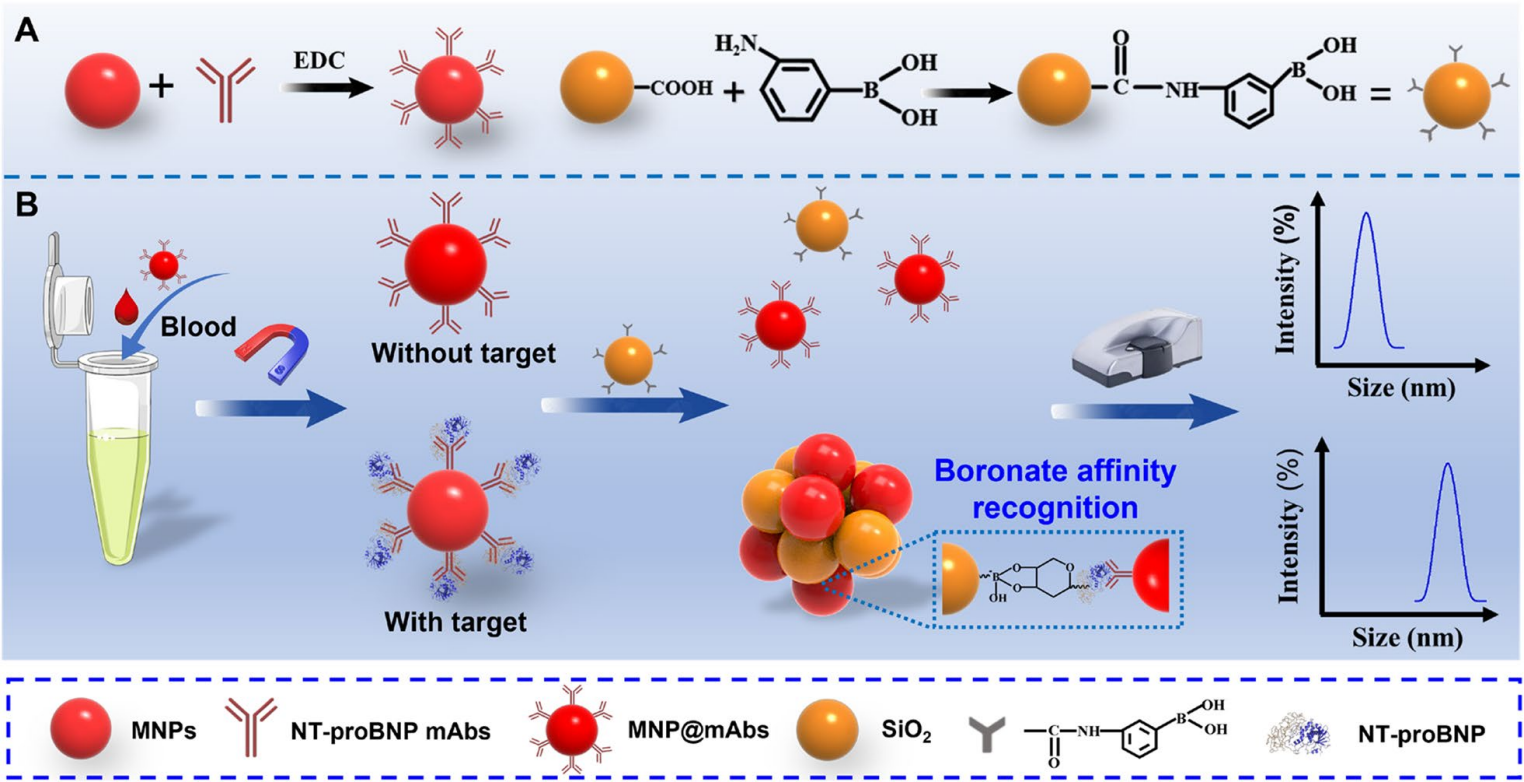
Schematic illustration of the developed DLS immunosensor platform for NT-proBNP detection by boronate affinity amplification. **A** Design and preparation of MNP@mAb conjugates and SiO_2_@PBA crosslinking agents in the detection system. **B** Schematic illustration of target recognition and capture by MNP@mAb probe, SiO_2_@PBA-mediated crosslinking signal amplification, and the result readout by DLS analyzer

As shown in Scheme 1A, the SiO_2_@PBA conjugates were prepared through the formation of amino linkage in the presence of EDC. In brief, 200 μL of SiO_2_ (50 mg mL^−1^) was added to 2 mL of 0.01 M PB (pH 6.0). After gentle stirring, 150 μL of PBA solution (50 mg mL^−1^) was added, in which PBA solution was prepared by dissolving 10 mg of 3-aminophenyl borate hydrochloride into 200 µL of 0.01 M PB (pH 8.0). Then, the pH of the mixed solution was adjusted to 6.5–7.0 by 10 M NaOH. After gentle stirring for 30 min at room temperature, 250 μg of EDC was added into the mixed solution. After adding EDC three times, the mixed solution was purified by centrifugation at 12,000 rpm for 15 min. After washed with PB (0.01 M, pH 7.4) three times, the resulting SiO_2_@PBA products were finally obtained.

### DLS immunosensor for NT-proBNP detection

About 6 μL of MNP@mAb (0.4 mg mL^−1^) solution was added into 200 µL of serum solution containing desired concentrations of NT-proBNP ranging from 0.01 pg mL^−1^ to 100 pg mL^−1^. After incubation at 37 °C for 5 min, the formed complex of MNP@mAb-NT-proBNP was collected under an external magnetic field for 10 min and washed thrice with 0.01 M PB (pH 7.4). Subsequently, the formed immunocomplex was then re-suspended in 800 μL of 0.01 M PB solution (pH 7.4) containing SiO_2_@PBA with the concentration of 4 μg mL^−1^. After incubation for 5 min, the D_H_ of the sample solution was measured on a DLS nanoparticle analyzer equipped with a green laser and an avalanche photodiode detector. The detection conditions were as follows: temperature 25 °C, detector angle 173°, incident laser wavelength 628 nm, and laser power 4.0. Data were analyzed and processed using a Malvern Zetasizer Nano application software. For each sample, three DLS analysis duplicates were carried out with approximately 12 runs and each run lasting 10 s at a scattering angle of 173°. The real-world applications of the proposed DLS immunosensor were conducted by an assay of NT-proBNP in serum, in which 1 μL of sample solution was added with 199 µL of 0.01 M PB solution (pH 7.4) and then subjected to DLS analysis.

## Results and discussion

### Working principle of the developed DLS immunosensor for NT-proBNP

Scheme 1 describes the working principle of the developed DLS immunosensor for the quantitative detection of NT-proBNP, wherein MNP@mAb was employed for magnetic enrichment of target analytes and DLS signal transduction, and SiO_2_@PBA was designed as crosslinkers to amplify the crosslinking aggregation of MNPs. When NT-proBNP was present in the sample solution, target glycoprotein was selectively captured by the MNP@mAb to form the immunocomplex of MNP@mAb-NT-proBNP. After washing to remove the supernatant, the immunocomplex was re-suspended in PB solution containing boronic acid crosslinkers, thereby inducing the MNP aggregation by the selective boronic acid ligand–*cis*-diol recognition between the SiO_2_@PBA and the glycoprotein. With the crosslinking aggregation of MNPs, the D_H_ of the solution will remarkably increase, which can be readily measured by DLS. Specifically, when the content of NT-proBNP is more, the MNP aggregate is larger, and the D_H_ of the solution is greater. By contrast, no obvious aggregation of MNPs was observed when the target NT-proBNP was absent, thus resulting in negligible changes in the D_H._ Therefore, the quantitative detection of glycoproteins in unknown samples can be achieved by recording the variation in the D_H_ of MNPs.

### Synthesis and characterization of MNP@mAb and SiO_2_@PBA

**Fig. 1:**
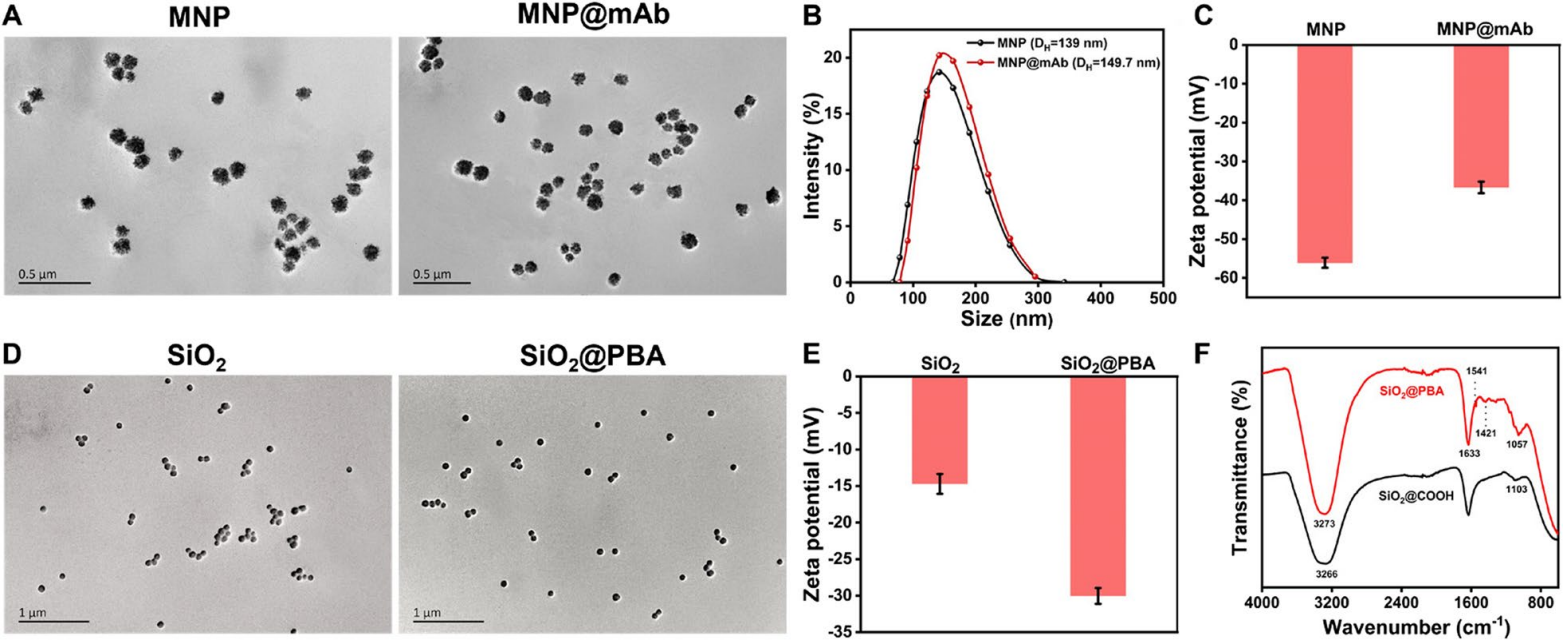
Characterization of the prepared MNP@mAb and SiO_2_@PBA conjugates. **A** TEM images of MNP and MNP@mAb. **B** The hydrodynamic diameter distribution of MNP and MNP@mAb. **C** The zeta potential of MNP and MNP@mAb. **D** TEM images of SiO_2_ and SiO_2_@PBA. **E** The zeta potential of SiO_2_ and SiO_2_@PBA. **F** The FTIR spectra of SiO_2_ (black curve) and SiO_2_@PBA (red curve)

The MNP@mAb conjugates were prepared through the formation of peptide linkage between the carboxyl group of MNPs and the amino group of anti-NT-proBNP mAb in the presence of EDC. The successful construction of the MNP@mAb was confirmed by TEM and DLS. As shown in Fig. 1A, the MNPs show uniform morphology and good monodispersity before and after conjugated with mAb. DLS measurement indicates an obvious increase in the D_H_ of MNPs from 139 to 149.7 nm after the conjugation of mAbs (Fig. 1B). Figure 1C exhibits the MNP@mAb has higher zeta potential of − 36.7 mV than that of unmodified MNPs (− 56.1 mV). These results suggest the successful conjugation of MNPs with mAbs. As shown in Scheme 1A, the SiO_2_@PBA conjugates were synthesized by a similar EDC-assisted covalent coupling method. The successful modification of PBA molecules on the surface of SiO_2_ was verified using TEM, DLS and FTIR. Figure 1D and Additional file 1: Fig. S1 reveals that no obvious changes in the morphology and monodispersity of SiO_2_ were observed after modified with the PBA. Figure 1E shows that the zeta potential of SiO_2_ reduced from − 15.0 to − 31.0 mV with the size increased from 114.4 to 124 nm when PBA molecules were modified onto the surface of SiO_2._ Further FTIR analysis of SiO_2_@PBA was compared with SiO_2_. As shown in Fig. 1F, FTIR spectra of SiO_2_ and SiO_2_@PBA present two characteristic peaks at 1057 cm^−1^ and 3273 cm^−1^, which correspond to the Si–O band and −OH, respectively [27]. In addition, the FTIR peaks of SiO_2_@PBA at 1633 cm^−1^, 1541 cm^−1^ and 1421 cm^−1^ correspond to the stretching vibration of a benzene ring skeleton, the peptide bond and −B(OH)_2_, respectively, which were not observed in SiO_2_ alone [28]. These findings prove that PBA molecules were successfully modified onto the surface of SiO_2_. The quantification of PBA on the surface of SiO_2_ was conducted and the details were shown in Additional file 1: Fig. S2.

### Confirmation of feasibility of the developed DLS immunosensor for NT-proBNP

**Fig. 2:**
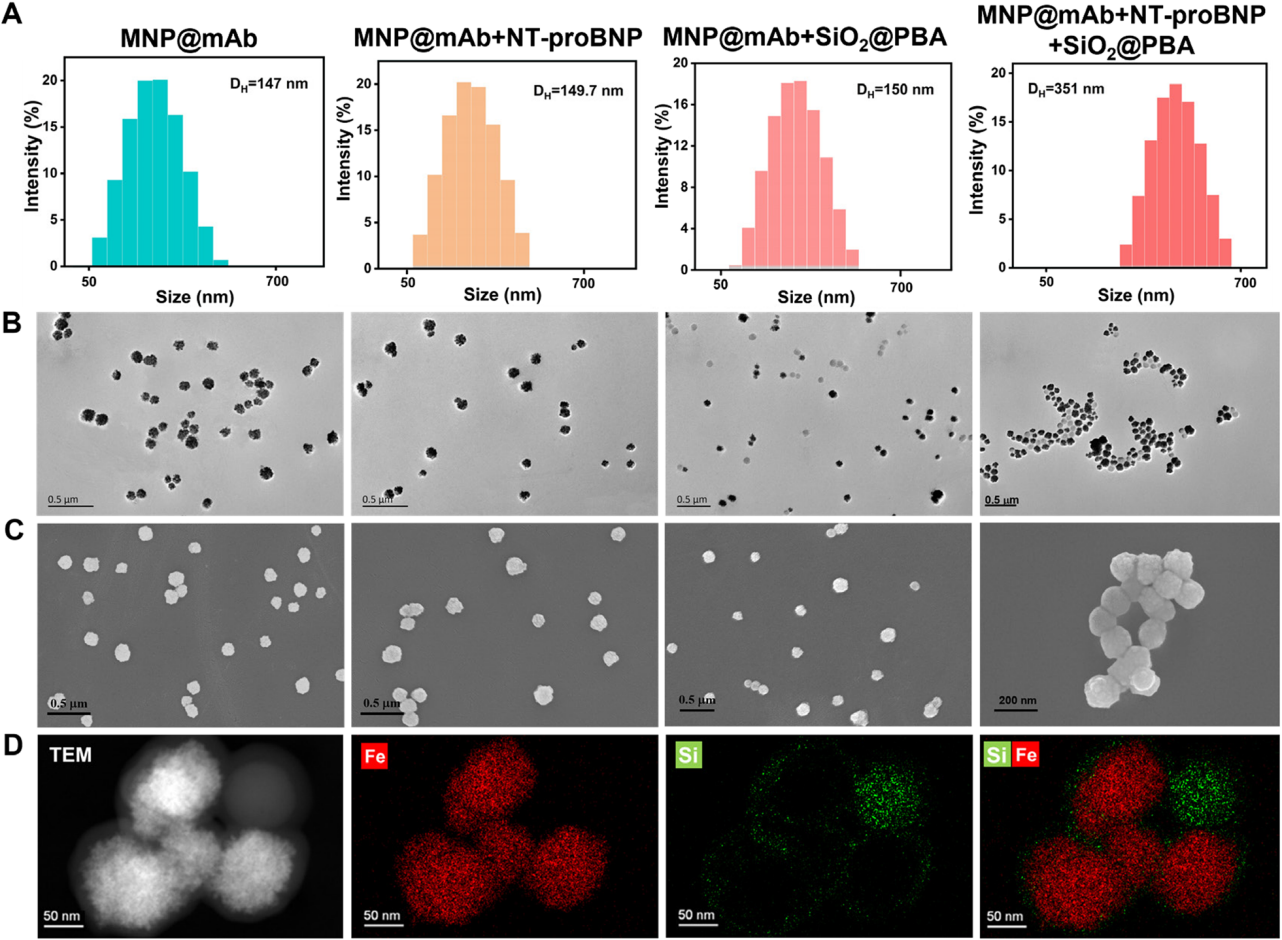
Verification of the developed DLS immunosensor for NT-proBNP detection by boronate affinity amplification. **A** Hydrodynamic diameter distribution, **B** TEM images, and **C** SEM images of MNP@mAb, MNP@mAb + NT-proBNP, MNP@mAb + SiO_2_@PBA, MNP@mAb + NT-proBNP + SiO_2_@PBA. **D** EDS mapping analysis of MNP@mAb + NT-proBNP + SiO_2_@PBA

The feasibility of the developed DLS immunosensor for the quantitative analysis of NT-proBNP was verified by conducting a series of control experiments, including (1) MNP@mAb, (2) MNP@mAb + NT-proBNP (20 pg mL^−1^), (3) MNP@mAb + SiO_2_@PBA, and (4) MNP@mAb + NT-proBNP (20 pg mL^−1^)  + SiO_2_@PBA. The formation of MNP aggreates caused by target NT-proBNP was monitored by DLS and TEM. The results in Fig. 2A and Additional file 1: Fig. S3 present that only the co-occurrences of target NT-proBNP and SiO_2_@PBA (denoted as MNP@mAb + NT-proBNP (20 pg mL^−1^) + SiO_2_@PBA) can result in a significant increase in the D_H_ of MNP@mAb from 147 to 351 nm. By contrast, two other groups, including MNP@mAb + NT-proBNP and MNP@mAb + SiO_2_@PBA, show negligible changes in the D_H_ compared with MNP@mAb alone. The reason for this phenomenon is that the presence of target NT-proBNP can specifically induce the MNP aggregation caused by SiO_2_@PBA, which are well demonstrated by SEM and TEM imaging (Fig. 2B, C). The formation of MNP aggreates caused by the SiO_2_@PBA was further confirmed by energy dispersive spectroscopy (EDS) mapping analysis (Fig. 2D) with the coexistence of Si and Fe in the aggregates. In addition, the results from magnetic relaxation switch sensing analysis prove the occurrence of boronate affinity reaction between SiO_2_@PBA and MNP@mAb in the presence of NT-proBNP (Additional file 1: Fig. S4). These observations verify the feasibility of the proposed boronate affinity amplified DLS immunosensor for ultrasensitively and specifically targeting NT-proBNP.

### Optimization of experimental conditions

**Fig. 3:**
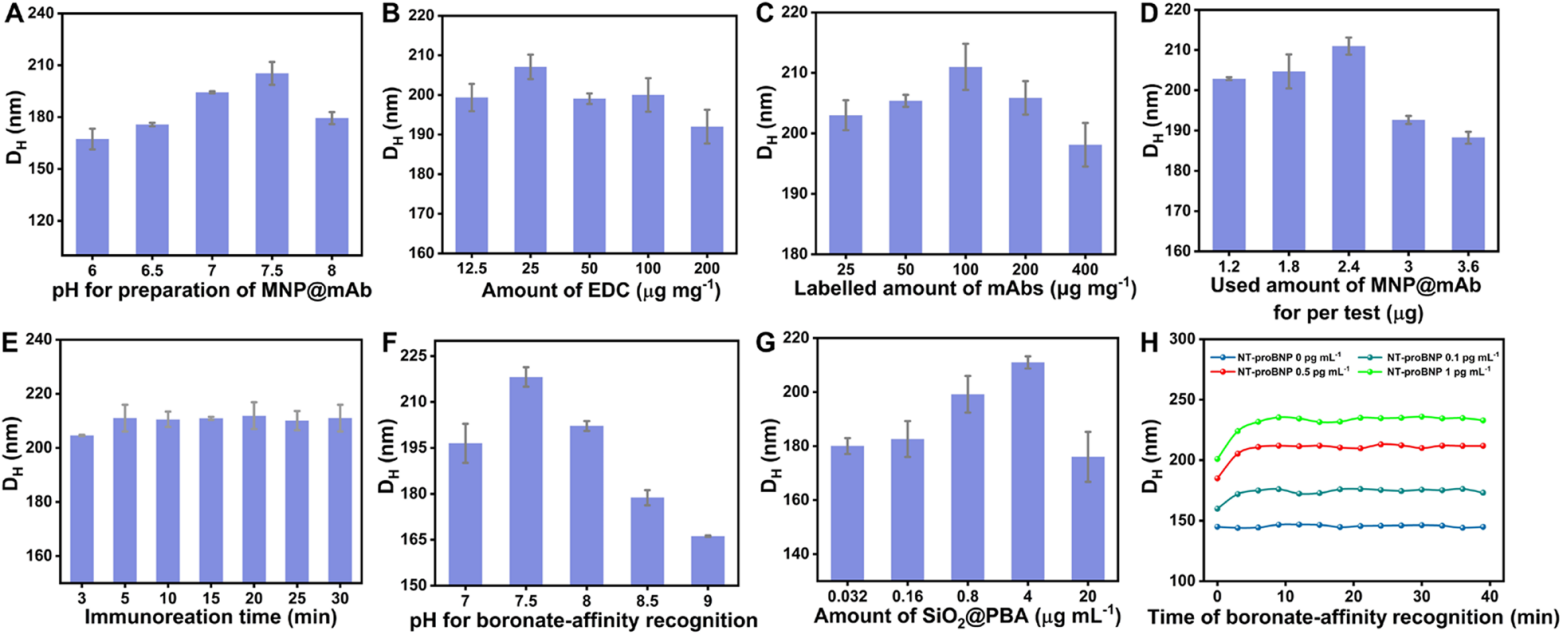
Parameter optimization. **A** pH value. **B** EDC amount. **C** The saturated labelling amount of mAbs for the preparation of MNP@mAb. **D** The used amount of MNP@mAb for per test. **E** The immunoreaction time of MNP@mAb for the capture of BNP from the sample solution. **F** The solution pH of boronate affinity recognition between NT-proBNP and SiO_2_@PBA. **G** The used amount of SiO_2_@PBA. **H** The reaction time of boronate affinity recognition between NT-proBNP and SiO_2_@PBA

To achieve the best analytical performance of the developed DLS immunosensor, several key parameters, such as the pH value, EDC amount, and labelled amount of mAbs for the preparation of MNP@mAb; the used amount of MNP@mAb for each test; the immunoreaction time of MNP@mAb and target NT-proBNP; the used amount of SiO_2_@PBA, pH value and incubation time for boronate affinity reaction between NT-proBNP and SiO_2_@PBA, were systematically studied. The optimized experimental conditions were evaluated by detecting the largest D_H_ change using DLS. The results in Fig. 3A–C indicate the optimal combinations of pH value, EDC amount, and labelled amount of mAbs for the preparation of MNP@mAb were 7.5, 25 μg mL^−1^, and 100 μg mg^−1^, respectively, wherein the MNP@mAb maintains the best bioactivities for the recognition and capture of target NT-proBNP, thus giving the maximal D_H_ values. Figure 3D show that the D_H_ gradually increased with increasing the used amount of MNP@mAb from 1.2 to 2.4 μg per test, and then obviously decreased with the further increase of MNP@mAb, which may be due to the presence of excess MNP@mAb to cause a reduction in the aggregate formation and size. The immunoreaction time between the MNP@mAb and the target NT-proBNP was further investigated to ensure the high sensitivity and reproducibility. Figure 3E shows that 5 min of immunoreaction time was necessary to result in the maximum D_H_ with high reproducibility. In addition, to further maximize the variation in the D_H,_ the boronate affinity recognition between the target glycoprotein and the boronic acid crosslinker is critical to control the crosslinking aggregation of MNPs for amplifying the DLS signal and improving the sensitivity. Figure 3F displays the effect of pH ranged from 7 to 9 on the MNP aggregation, and the results show the greatest D_H_ value at pH 7.5. With increasing the amount of SiO_2_@PBA from 0.032 to 4 μg mL^−1^, the D_H_ value increased gradually from 224.7 to 259.6 nm (Fig. 3G). However, the D_H_ value decreased obviously as the concentration of crosslinking agents continued to increase to 20 μg mL^−1^. The possible reason is that the excess crosslinker could block the remaining *cis*-diol sites on the glycoprotein molecules and in turn inhibit the MNP aggregation, thus giving rise to a decreased D_H._ Figure 3H shows that at all NT-proBNP concentrations, the D_H_ value reached a constant after 5 min reaction of the glycoprotein and the SiO_2_@PBA, suggesting 5 min was enough to allow sensitive and reproducible DLS signal transduction for reliable quantitative analysis.

### Performance evaluation of the amplified DLS immunosensor

**Fig. 4:**
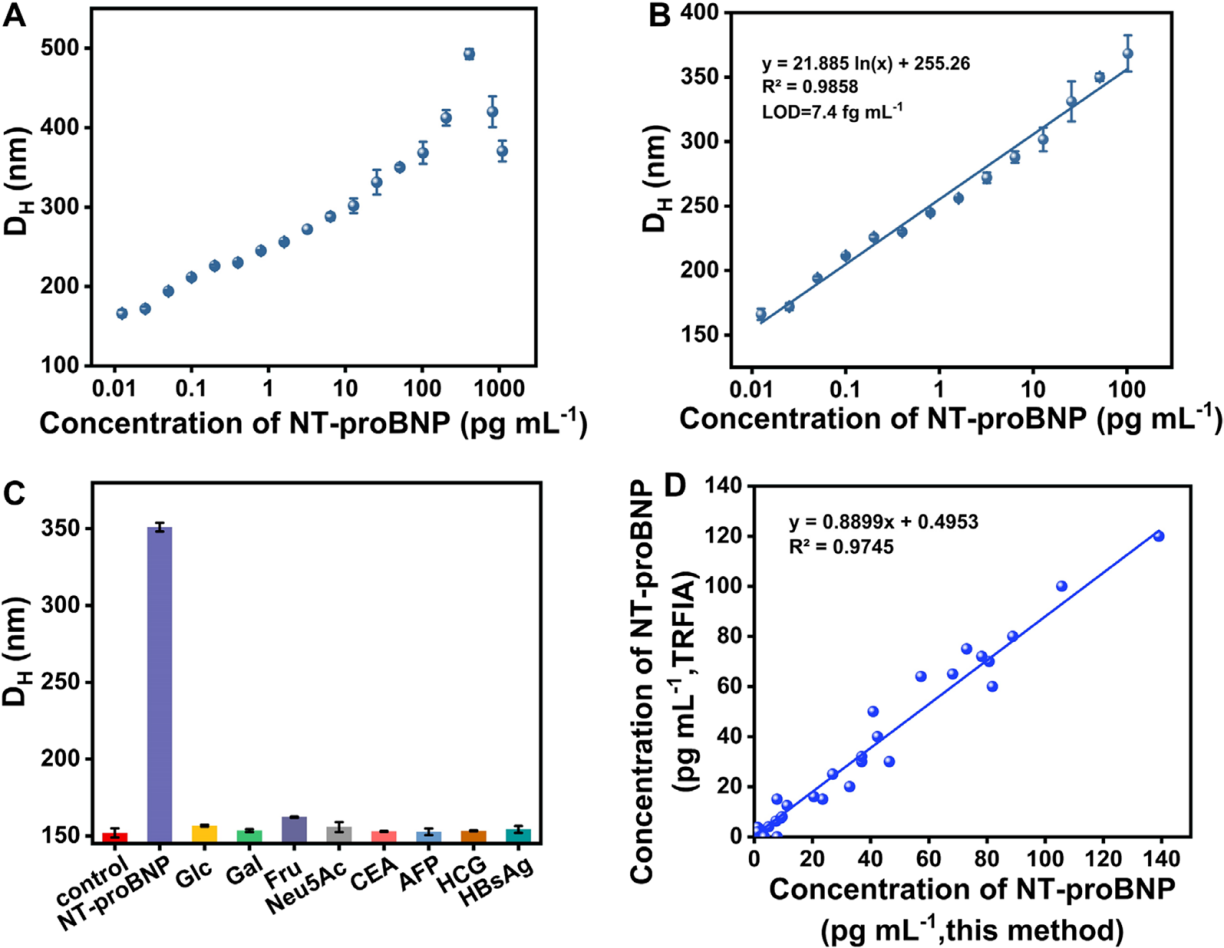
**A** The dose–response relationship between the D_H_ and the NT-proBNP concentration ranged from 0.012 to 1100 pg mL^−1^. **B** Standard curve for NT-proBNP detection with the concentration ranged from 0.012 to 100 pg mL^−1^. **C** Specificity evaluation for NT-proBNP (20 pg mL^−1^) by analyzing other common non-target proteins and saccharides, including Glc (5 mg mL^−1^), Gal (5 mg mL^−1^), Fru (5 mg mL^−1^), Neu5Ac (1 mg mL^−1^), CEA (1 ng mL^−1^), AFP (1 ng mL^−1^), HCG (500 mIU mL^−1^), and HBsAg (1 ng mL^−1^). **D** A correlation analysis between the detection results obtained from the developed DLS immunosensor and the TRFIA in detecting 30 NT-proBNP-positive serum samples

The signal response of this DLS immunosensor against different concentrations of NT-proBNP was examined under the developed conditions. As shown in Fig. 4A, the D_H_ value gradually increased with the NT-proBNP concentration ranging from 0.012 to 1100 pg mL^−1^. Figure 4B shows an excellent linearity between the D_H_ value and the logarithm of the NT-proBNP concentration (0.012–100 pg mL^−1^). The linear regression equation is described as: *y* = 21.885ln*x* + 255.26, with the correlation coefficient of 0.9858. The limit of detection (LOD) was calculated to be 7.4 fg mL^−1^ according to the mean plus three-fold standard deviations of the detected concentrations from twenty blank samples [21]. This LOD value is much lower than other previously reported immunoassays for NT-proBNP detection (Table 1), demonstrating that our immunosensor has better sensitivity. Notably, the total time to complete a test is less than 20 min, which is comparable to that of the most widely used POC device of lateral flow assay and obviously shorter than other reported immunoassays. The ultrahigh sensitivity and fast response time are ascribed to the boronate affinity-based multivalent recognition between crosslinkers and target glycoproteins and the magnetic enrichment of targets from the complex sample. Additionally, an obvious hook effect was observed at the NT-proBNP concentration of over 200 pg mL^−1^, indicating that additional sample dilution was needed to ensure accurate quantification of NT-probNP and avoid false results when the concentration of NT-probNP exceeds the hook point.

**Table 1:** Comparison of analytical performances of the proposed method with other reported analytical technologies in detecting NT-proBNP

Detection method	LOD (pg mL^−1^)	Linear range (pg mL^−1^)	Time (min)	Refs
LFIA	10	50 to 1200	/	[29]
ELISA	400	970 to 23,100	120	[30]
ECL	6	20 to 100,000	85	[6]
	3	5 to 4000	18	[31]
PEC	0.32	0.8 to 45,000	150	[32]
	3.7	10 to 50,000	80	[8]
DLS	0.0074	0.012 to 100	20	This work

DLS: dynamic light scattering; LFIA: lateral flow immunoassay; ELISA: enzyme linked immunosorbent assay; ECL: electrochemiluminesce; PEC: photoelectrochemical

The specificity of this developed DLS immunosensor was characterized by using a series of common interfering glycoproteins, including carcinoembryonic antigen (CEA), alpha fetoprotein (AFP), human chorionic gonadotropin (HCG), and hepatitis B virus antigen (HBsAg), as well as monosaccharides such as glucose (Glc), galactose (Gal), fucose (Fuc), and *n*-acetylneuraminic acid (Neu5Ac). Figure 4C indicated that only NT-proBNP can provide an apparent signal value, while other interferents show negligible changes compared with the negative control. These results indicate that the designed DLS immunosensor can specifically distinguish target glycoprotein from other interfering substances because of the specific immunorecognition between NT-proBNP and its captured antibody.

The accuracy and precision of the proposed DLS immunosensor for the quantitative detection of NT-proBNP were further investigated by measuring the intra- and inter-assay recoveries. As indicated in Additional file 1: Table S1, the intra-assay recoveries ranged from 89.8 to 102.1% with the coefficient of variation (CV) of 0.3% to 10.8%, while the inter-assay recoveries varied from 80.8 to 104.1% with the CV of 3.9 to 7.9%. These results indicate an acceptable accuracy and precision for quantitative determination of NT-proBNP in complex samples.

The reliability and practicability of this DLS immunosensor in actual samples were demonstrated by the detection of NT-proBNP in human serum. Forty NT-proBNP-positive serum samples collected from the First Affiliated Hospital of Nanchang University were simultaneously analyzed by the developed immunosensor and the clinical routine timed-resolved fluoroimmunoassay (TRFIA). For our method, 1 μL of serum samples were diluted with 199 µL of 0.01 M PB solution (pH 7.4) and then quantified, while the analysis of NT-proBNP using the TRFIA was performed according to the manufacturer’s instructions. The test results obtained by these two approaches were then compared. As indicated in Additional file 1: Table S2, among these 36 samples, 34 samples were tested with NT-proBNP as detected by our proposed DLS immunosensor method. By contrast, 30 samples were tested NT-proBNP positive by the TRFIA method. These results showed that compared with the routinely used TRFIA, the newly developed DLS immunosensor can detect lower concentrations NT-proBNP, which is attributed to its higher sensitivity (LOD, 1.5 pg mL^−1^) than that of the TRFIA method (70, pg mL^−1^). In addition, 2 samples were simultaneously detected NT-proBNP negative by these two methods. Figure 4D and Additional file 1: Fig. S5 exhibit that the detection results of the proposed method are broadly in line with those of TRFIA with a good linear correlation of 0.9745, proving the feasibility of the amplified DLS immunosensor for real-world applications in complex sample matrix.

## Conclusion

In conclusion, we successfully developed an ultrasensitive immunosensor for the simple and rapid detection of NT-proBNP in complex samples by coupling boronate affinity-mediated crosslinking aggregation with DLS transduction. For this purpose, SiO_2_@PBA was designed as the crosslinking agent to control the MNP aggregation and amplify the DLS signal in the presence of target NT-proBNP, thus contributing to the detection sensitivity. Thanks to the multivalent and rapid boronate affinity reaction, this proposed DLS immunosensor has the advantages of high sensitivity (7.4 fg mL^−1^), short response time (20 min), and small sample consumption (1 μL), exhibiting great potential for POC use. The analytical performances of this immunosensor were demonstrated in selectivity, accuracy, and practicability. Moreover, the feasibility and reliability of this approach for real-world applications was characterized by an assay of trace NT-proBNP in human serum, which was further corroborated by the clinical routine TRFIA. Briefly, the boronate affinity-amplified DLS immunosensing platform provides a promising analytical tool for POC detection of *cis*-diol-containing compounds, such as glycoproteins.

## Supplementary Information

**Additional file 1.** Additional figures and tables.

## Data Availability

All data used to support the findings of this study are available from the corresponding author upon request.
