# Supplementary material for: Ultrasensitive dynamic light scattering immunosensing platform for NT-proBNP detection using boronate affinity amplification
Source: J Nanobiotechnology. 2022 Jan 6;20:21. doi: 10.1186/s12951-021-01224-5 (PMC8740487; doi:10.1186/s12951-021-01224-5)
Supplement: Supplementary file 1 — Additional file 1. Additional figures and tables. [file 12951_2021_1224_MOESM1_ESM.docx]

**Supporting Information**

**Ultrasensitive dynamic light scattering immunosensing platform for NT-proBNP detection using** **boronate affinity amplification**

Jiaqi Hu^a,c#^, Lu Ding^b#^, Jing Chen ^a,c^, Kang Zhu^a,c^, Qian Guo^a,c^, Xiaolin Huang^*a,c^, and Yonghua Xiong^a,c^

^a^ State Key Laboratory of Food Science and Technology, School of Food Science and Technology, Nanchang University, Nanchang 330047, P.R. China;

^b^ Hypertension Research Institute of Jiangxi Province, Department of Cardiology, The First Affiliated Hospital of Nanchang University, Nanchang, Jiangxi 330006, P. R. China;

^c^ Jiangxi-OAI Joint Research Institute, Nanchang University, Nanchang 330047, P.R. China

^#^ These authors contributed equally to this work.

***Correspondence to:**

Dr. and Prof. Xiaolin Huang

School of Food Science and Technology, Nanchang University, Nanchang 330047, PRC

**E-mail:** [hxl19880503@163.com](mailto:hxl19880503@163.com) and [xiaolin.huang@ncu.edu.cn](mailto:xiaolin.huang@ncu.edu.cn)


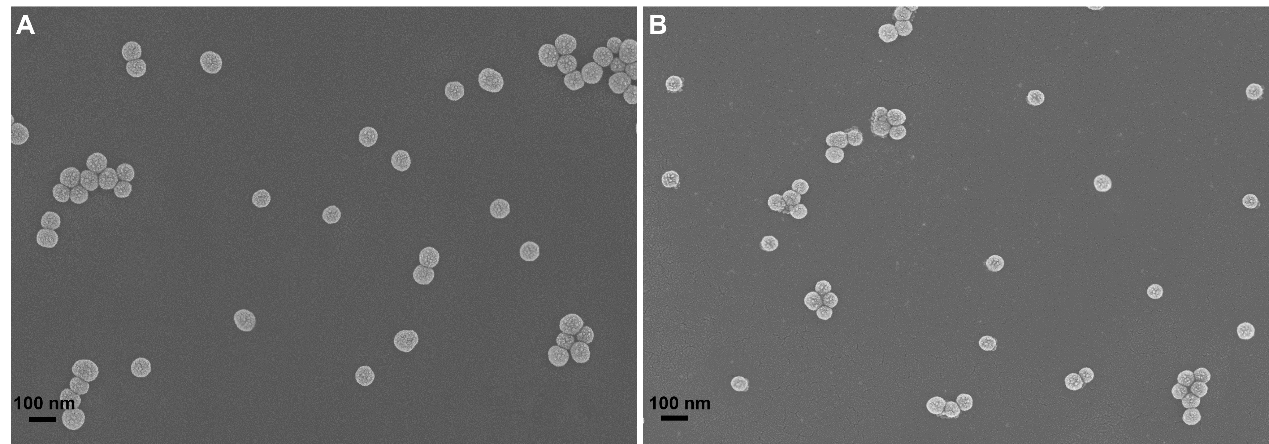


**Figure S1.** SEM images of (A) SiO_2_ and (B) SiO_2_@PBA.


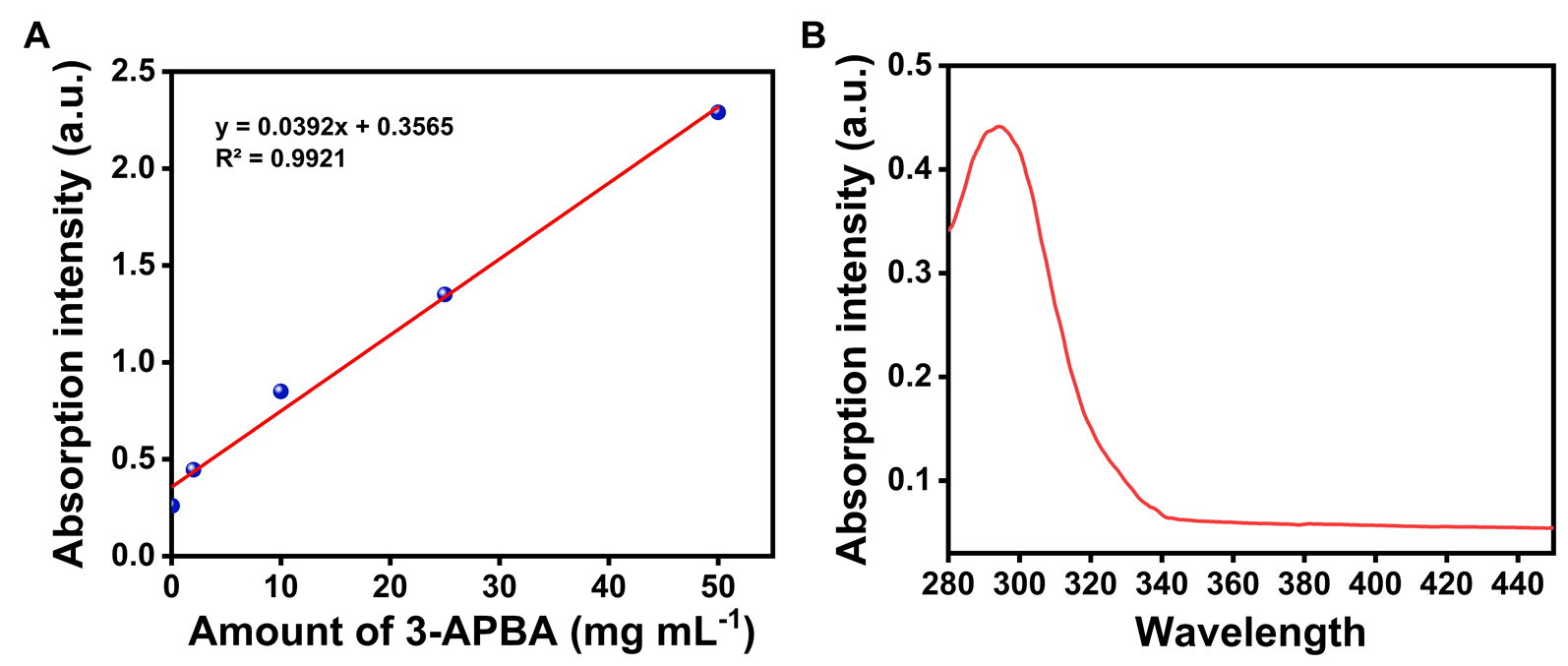


**Figure S2.** A.The dose–response relationship between the UV absorption intensity and the 3-APBA concentration. B. UV absorption spectrum of 3-APBA in the supernatant.

**Results:** we first established a standard curve based on the different concentrations of 3-APBA in PB 6.0 solution, and the linear regression equation is described as: y = 0.0392x + 0.3565, with the correlation coefficient of 0.9921. Then, the 3-APBA was conjugated with the carboxyl groups modified SiO_2_. After the separation of SiO_2_@PBA conjugate *via* centrifugation, the unreacted 3-APBA in the supernatant was determined by the UV absorption spectrum (Figure S2). The amount of 3-APBA on per mg of SiO_2_ was calculated at 0.574 mg.

**Reference:**

1. Kur K, Przybyt M, Miller E. Study of 3-amino phenylboronic acid interactions with selected sugars by optical methods[J]. Journal of Luminescence, 2017, 183: 486-493.


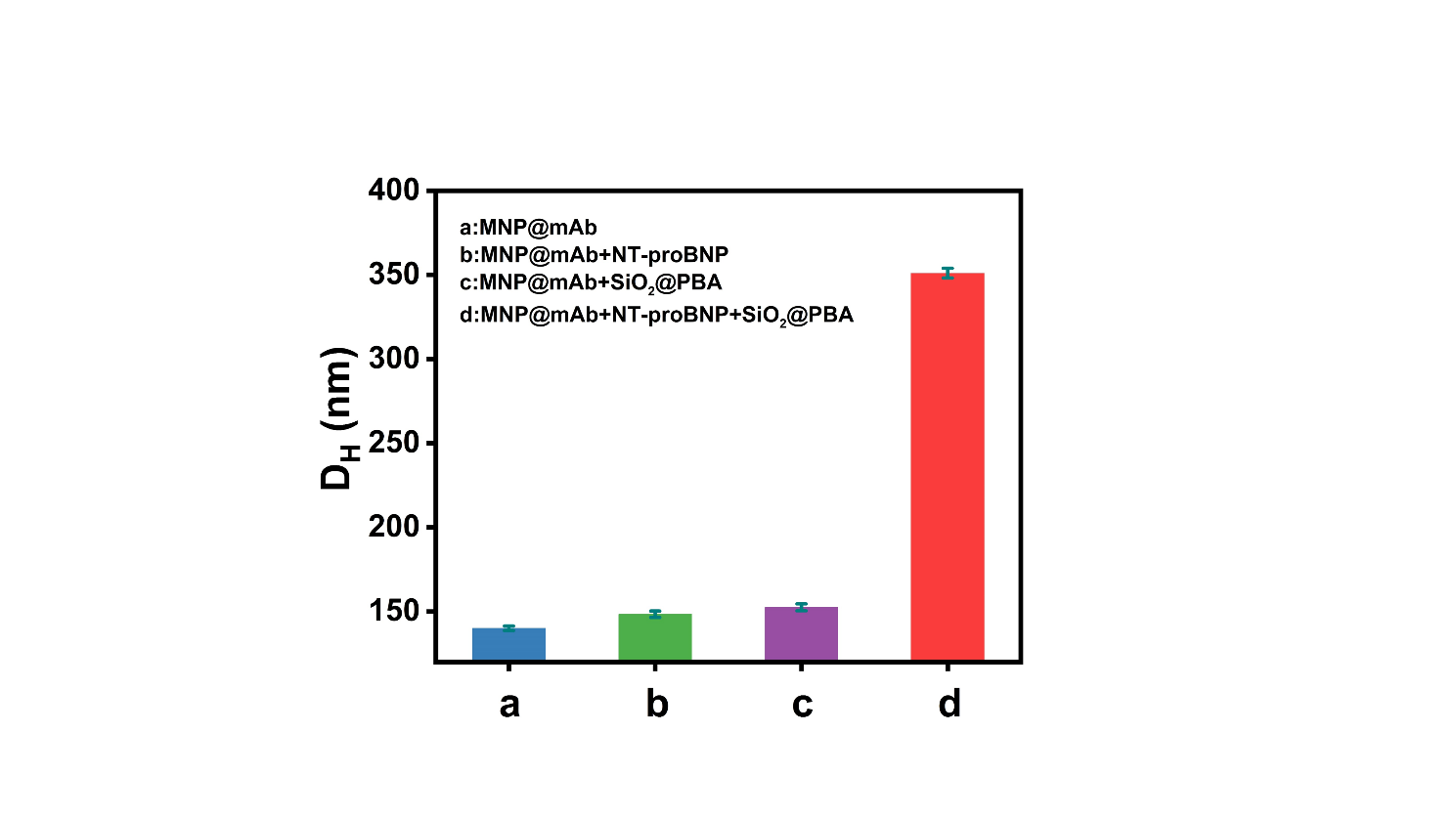


**Figure S3.** DLS analysis of MNP@mAb, MNP@mAb + NT-proBNP, MNP@mAb + SiO_2_@PBA, MNP@mAb + NT-proBNP + SiO_2_@PBA.


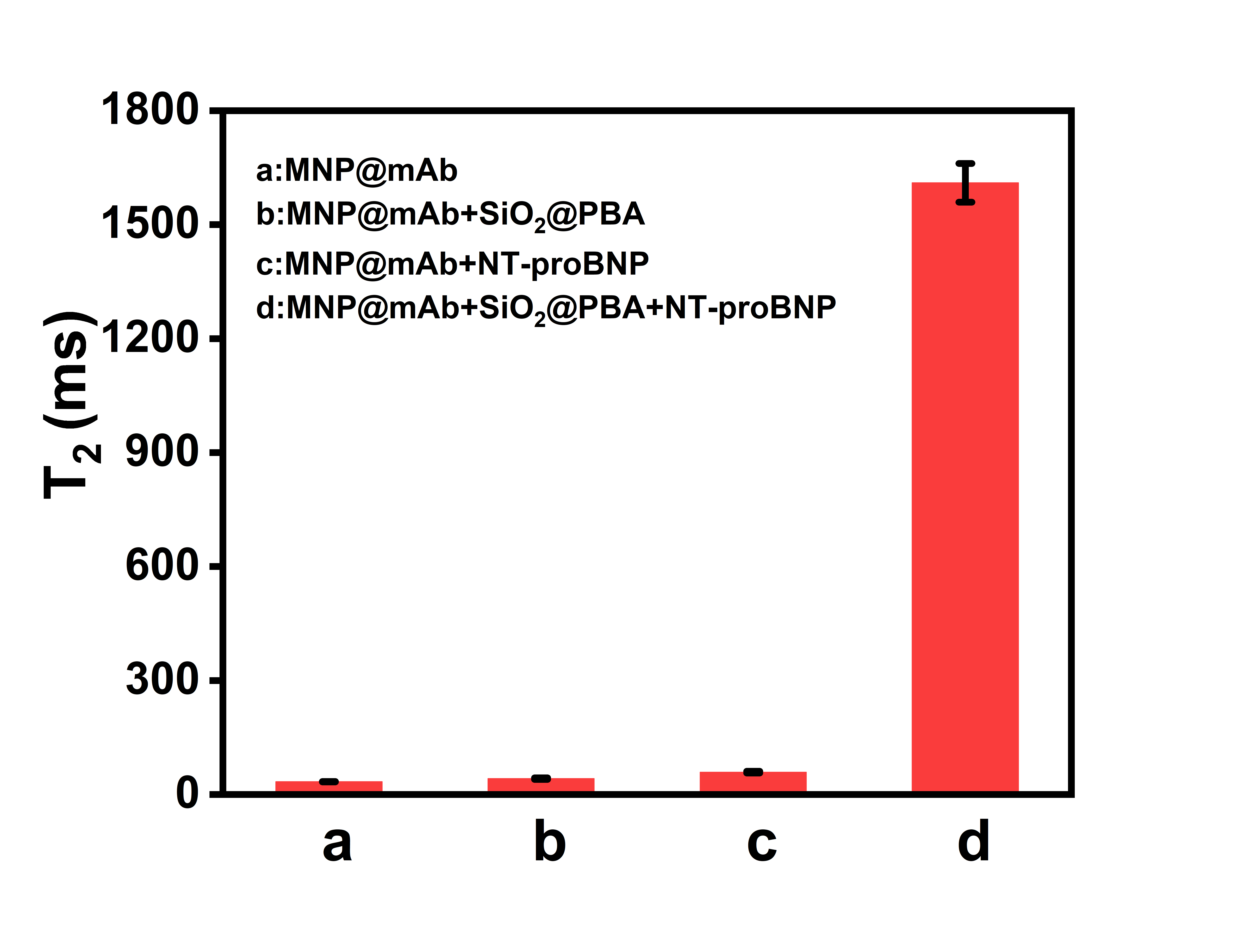


**Figure S4.** MRS analysis of MNP@mAb, MNP@mAb+NT-proBNP, MNP@mAb+SiO_2_@PBA, MNP@mAb+NT-proBNP+SiO2@PBA.

**Results:** Magnetic relaxation switch (MRS) sensing was adopted to verify the aggregates between MNP@mAb + SiO_2_@PBA in the presence of NT-proBNP. According to the difference of the size of MNPs, the lateral relaxation time (T_2_) caused by the aggregation of MNPs shows the increased (MNP size of ≥ 100 nm) or decreased (MNP size of 1-100 nm) signals. Owing to the MNP size of l47 nm used in this work, Figure S4 showed a remarkably increased T_2_ signal in the group of MNP@mAb+SiO_2_@PBA+NT-proBNP compared with other control groups, which further demonstrated that the SiO_2_@PBA could trigger the aggregation of MNP@mAb in the presence of NT-proBNP.

**Reference:**

1. Zhao J, Wang Z, Chen Y, et al. Horseradish peroxidase-catalyzed formation of polydopamine for ultra-sensitive magnetic relaxation sensing of aflatoxin B1. Journal of Hazardous Materials, 2021: 126403.

2. Koh I, Hong R, Weissleder R, et al. Sensitive NMR sensors detect antibodies to influenza. Angewandte Chemie, 2008, 120(22): 4187-4189.

3. Chen Y, Zou M, Li Y, et al. An immunosensor based on magnetic relaxation switch and polystyrene microparticle-induced immune multivalency enrichment system for the detection of *Pantoea stewartii subsp. stewartii*. Biosensors and Bioelectronics, 2013, 43: 6-11.


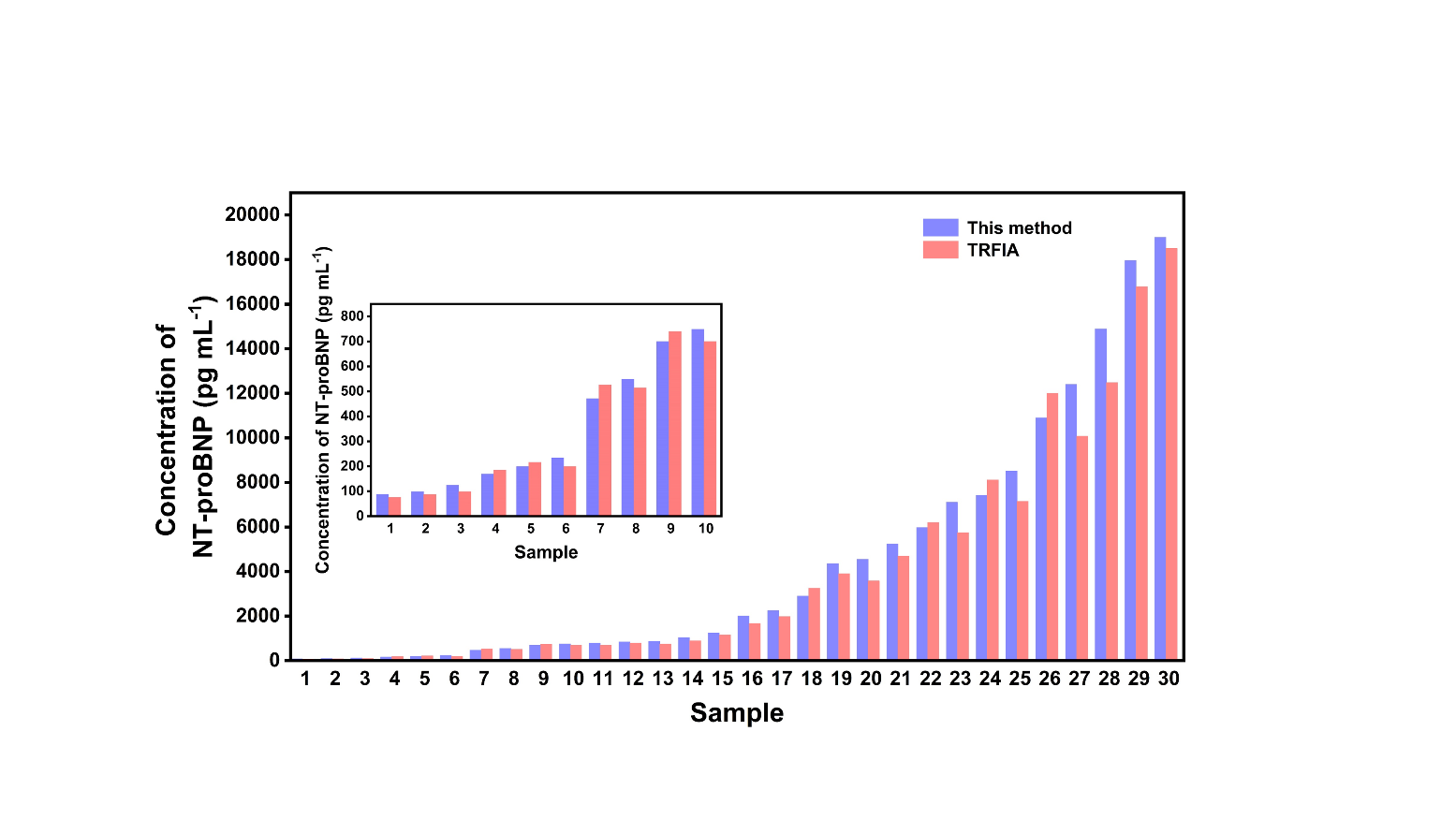


**Figure S5.** A correlation analysis between the detection results obtained from the developed DLS immunosensor and the TRFIA in detecting 30 NT-proBNP-positive serum samples.

**Table S1.** Accuracy and precision of the developed DLS immunosensor in serum samples.

| NT-proBNP concentration  （pg mL^−1^） | Inter-assay precision | | | Intra-assay precision | | |
| --- | --- | --- | --- | --- | --- | --- |
|  | Mean^a^ | Recovery  (%) | CV  (%) | Mean^b^ | Recovery  (%) | CV  (%) |
| 0.025 | 0.023 | 92.0 | 6.8 | 0.023 | 92.0 | 7.9 |
| 0.1 | 0.097 | 97.0 | 10.8 | 0.099 | 99.0 | 3.9 |
| 0.5 | 0.51 | 102.1 | 0.3 | 0.52 | 104.1 | 5.6 |
| 5 | 4.78 | 95.6 | 3.2 | 4.04 | 80.8 | 4.6 |
| 50 | 50.6 | 101.2 | 8.6 | 47.6 | 95.2 | 6.3 |

^a^ Mean value of four replicates at each diluted concentration.

^b^ Assay was completed every day for three days continuously.

**Table S2.** A correlation analysis for AFP detection in 36 clinical serum samples between the DLS immunosensor and the TRFIA method.

| Sample | TRFIA  (pg mL^-1^) | Our method (pg mL^-1^) | Sample | TRFIA  (pg mL^-1^) | Our method (pg mL^-1^) |
| --- | --- | --- | --- | --- | --- |
| 1 | 75.8 | 88.1 | 19 | 8114.1 | 7411.9 |
| 2 | 185.1 | 168.7 | 20 | 4695.4 | 5240.1 |
| 3 | － | 15.9 | 21 | 97.8 | 123.5 |
| 4 | 87.1 | 98.4 | 22 | － | － |
| 5 | 1160 | 1246.8 | 23 | 1680.1 | 2018.4 |
| 6 | 893.8 | 1036.4 | 24 | 85.4 | 90.8 |
| 7 | 745.9 | 870.9 | 25 | 102.4 | 99.1 |
| 8 | － | － | 26 | 205.9 | 194.2 |
| 9 | 216.2 | 198.6 | 27 | － | 35.1 |
| 10 | 526.1 | 474.2 | 28 | 214.7 | 200.7 |
| 11 | 8114.5 | 7412.3 | 29 | 5740.6 | 7103.8 |
| 12 | － | 24.8 | 30 | 86.5 | 92.6 |
| 13 | 3250.8 | 2904.1 | 31 | 514.9 | 547.9 |
| 14 | 1980.1 | 2248.3 | 32 | 208.4 | 189.7 |
| 15 | 738.8 | 700.4 | 33 | 16784.1 | 17950.4 |
| 16 | - | 50.1 | 34 | 12480.7 | 14889.1 |
| 17 | 3907.8 | 4354.8 | 35 | 7152.4 | 8512.1 |
| 18 | 6200.2 | 5969.5 | 36 | 3578.1 | 4550.1 |
